# Management of Great Saphenous Vein and Inferior Vena Cava Leiomyosarcomas: Two Surgical Case Reports and Literature Review

**DOI:** 10.3390/jcm15041636

**Published:** 2026-02-21

**Authors:** Patrik Buzgǎu, Emil-Marian Arbănași, Claudiu Constantin Ciucanu, Réka Bartus, Eliza-Mihaela Arbănași, Adrian Vasile Mureșan, Eliza Russu, Marius-Alexandru Beleaua, Emőke Horváth, Luca Tirloni, Matteo Risaliti, Ilenia Bartolini, Gian Luca Grazi

**Affiliations:** 1 Department of Anatomy, George Emil Palade University of Medicine, Pharmacy, Science and Technology of Targu Mures, 540139 Targu Mures, Romania; patrik.buzgau@umfst.ro; 2 Department of Vascular Surgery, George Emil Palade University of Medicine, Pharmacy, Science and Technology of Targu Mures, 540139 Targu Mures, Romania; claudiu.ciucanu@umfst.ro (C.C.C.); reka.kaller@umfst.ro (R.B.); adrian.muresan@umfst.ro (A.V.M.); eliza.russu@umfst.ro (E.R.); 3 Clinic of Vascular Surgery, Mures County Emergency Hospital, 540136 Targu Mures, Romania; 4 Regenerative Medicine Laboratory, Centre for Advanced Medical and Pharmaceutical Research (CCAMF), George Emil Palade University of Medicine, Pharmacy, Science and Technology of Targu Mures, 540139 Targu Mures, Romania; arbanasi.eliza@gmail.com; 5 Doctoral School of Medicine and Pharmacy, George Emil Palade University of Medicine, Pharmacy, Science and Technology of Targu Mures, 540139 Targu Mures, Romania; 6 Department of Pathology, George Emil Palade University of Medicine, Pharmacy, Science and Technology of Targu Mures, 540139 Targu Mures, Romania; marius.beleaua@umfst.ro (M.-A.B.); emoke.horvath@umfst.ro (E.H.); 7 Hepatopancreatobiliary Surgery Unit, Careggi University Hospital, Largo Brambilla 3, 50134 Florence, Italy; luca.tirloni@unifi.it (L.T.); risalitim@aou-careggi.toscana.it (M.R.); ilenia.bartolini@gmail.com (I.B.); gianluca.grazi@unifi.it (G.L.G.); 8 Department of Experimental and Clinical Medicine, University of Florence, 50121 Florence, Italy

**Keywords:** vascular leiomyosarcoma, IVC, vascular surgery, GSV, open surgery, case report

## Abstract

**Background**: Vascular leiomyosarcoma (LMS) is an exceptionally rare and aggressive soft tissue sarcoma arising from the smooth muscle cells of the vascular wall. They account for approximately 0.5–2% of adult soft-tissue sarcomas and are the most frequent primary malignancy of vascular origin. Among venous sites, the inferior vena cava (IVC) is the most frequently involved, accounting for more than half of reported vascular LMS cases, with rarer occurrences in peripheral veins, including the internal saphenous vein and the external iliac vein. **Case Presentation**: We report a case series comprising two distinct presentations of vascular LMS involving the internal saphenous vein and the inferior vena cava, respectively. Each case highlights unique clinical manifestations, radiologic features, histopathologic diagnosis, and therapeutic challenges inherent to the involved vascular territory. Surgical resection with clear margins was the primary treatment modality, complemented by adjuvant therapies tailored according to tumor grade and extent. **Literature Review**: An updated literature review contextualizes these findings, detailing epidemiology, diagnostic challenges, prognostic factors, and current management approaches. It emphasizes the rarity of leiomyosarcomas originating from major venous pathways and highlights variability in clinical presentation, tumor size, growth patterns, and outcomes. Achieving complete surgical removal with negative margins continues to be the primary treatment goal and the most significant prognostic factor. **Conclusions**: Given the paucity of cases, our series contributes valuable insights into the clinical spectrum and multidisciplinary approach necessary for optimal outcomes in vascular LMS. Early recognition and aggressive treatment remain paramount to improving survival in this rare malignancy.

## 1. Introduction

Soft tissue sarcomas (STS) constitute a rare and heterogeneous group of malignant neoplasms that originate from mesenchymal tissues, encompassing connective components such as muscle, adipose tissue, blood vessels, nerves, and fibrous tissues [1]. Accounting for less than 1% of all adult malignancies, STS encompasses over 100 distinct histological subtypes, each characterized by unique biological behaviors and variable responses to therapeutic interventions [1].

Vascular leiomyosarcomas (LMS) are exceptionally rare malignant variants arising from the smooth muscle cells of the tunica media. They represent approximately 0.5–2% of adult soft tissue sarcomas and constitute the most frequent primary malignancy of vascular origin. Notably, venous structures are affected far more often than arteries, with a reported predilection ratio of nearly five to one [2,3]. Among venous sites, the inferior vena cava (IVC) is the most frequently involved location, accounting for more than half of reported vascular LMS cases [4,5] with rarer occurrences reported in peripheral veins including the internal saphenous vein and the external iliac vein. Leiomyosarcomas of the great saphenous vein are particularly rare, with around 56 cases documented globally [6], often presenting with indolent, painless swelling that can be misdiagnosed as superficial venous thrombosis.

This case series aims to describe a rare presentation of leiomyosarcoma involving multiple major venous structures, emphasizing its clinical features, diagnostic complexities, and treatment strategies. The scope includes a comprehensive literature review to examine the epidemiology, pathological characteristics, and management approaches of leiomyosarcomas originating from large veins, providing valuable insights into their multifocal vascular involvement. This research seeks to enhance understanding of these uncommon tumors and support improved diagnostic and therapeutic decision-making in similar complex cases, being prepared in accordance with the CARE (CAse REport) guidelines for reporting clinical cases. All relevant clinical information, including patient history, diagnostic assessment, therapeutic interventions, and follow-up outcomes, is presented in accordance with the CARE checklist to ensure transparency and completeness.

## 2. Overview of Leiomyosarcomas in Major Veins

### 2.1. Leiomyosarcomas in the IVC

Approximately 700 cases of primary IVC leiomyosarcoma have been reported in the medical literature to date [7,8]. An international IVC leiomyosarcoma registry was established in 1992, initially recording around 300 cases. Over time, additional literature reviews and case reports have increased the total number of reported cases to nearly 700. Earlier reviews documented around 218 cases until 1996, and continuous reporting over the years has nearly tripled this number. The presentation of primary leiomyosarcomas of the IVC typically occurs with tumors of considerable size, attributable to the indolent and frequently asymptomatic course during the initial stages of the disease. Consequently, diagnosis is often established when tumors have reached more advanced dimensions. According to multiple recent studies and case series, the average tumor size at presentation ranges from approximately 8.5 cm to 12 cm [8,9]. Such tumors portend a poor prognosis due to several interrelated factors and frequently present at an advanced stage, characterized by substantial size and extensive local invasion involving adjacent anatomical structures such as the liver, renal veins, or even extension into the right atrium [8,10,11]. The lesion can be categorized into three levels based on its anatomical relationship to the hepatic and renal veins [12]. Zone I corresponds to the infrarenal segment of the IVC, Zone II encompasses the segment between the hepatic and renal veins, and Zone III includes the portion extending from above the hepatic veins to the right atrium. Inferior vena cava leiomyosarcomas most commonly arise in the middle segment (Zone II) [13]. Such involvement complicates achieving complete surgical resection with negative margins, which is critical for improved survival outcomes [2,13]. Consequently, achieving an R0 resection remains a formidable challenge, often necessitating complex multivisceral surgical approaches that increase perioperative morbidity [2,4].

### 2.2. Leiomyosarcomas in the Great Saphenous Vein

Great saphenous vein leiomyosarcomas (GSV-LMS) are very rare vascular tumors that often present with atypical, slow-growing clinical features. This frequently results in misdiagnosis as superficial venous thrombosis or other benign conditions [6,14]. Reported tumor sizes for GSV-LMS vary widely, from 1 cm to 17 cm. On average, tumor size is about 4.9 cm, based on intraoperative measurements from clinical cases. Many tumors are detected as masses between approximately 2 cm and over 10 cm. The size variation reflects differences in presentation timing since these tumors tend to grow slowly and are often diagnosed late due to their nonspecific symptoms [6,14,15,16].

The clinical course of GSV-LMS is often slow and aggressive, with risks of local recurrence and distant metastases, particularly to the lungs [6]. The prognosis appears to be mainly influenced by tumor location, especially through the extent of surrounding tissue involvement. Tumors in the thigh or groin tend to be more extensive, with a higher chance of local invasion or obstruction, which can lead to poorer clinical outcomes. Lesions near major venous junctions, such as the saphenofemoral junction, are associated with more severe symptoms, including limb edema, pain, venous obstruction, and recurrent or atypical thrombosis, as well as increased complication risks due to involvement of key venous structures. Additionally, the growth pattern—whether intracavitary, extracavitary, or mixed—affects prognosis, with mixed patterns possibly indicating more aggressive disease. Larger, proximally located tumors usually correspond to advanced stages at diagnosis and a higher risk of metastasizing, especially to the lungs, which negatively impacts survival [14,15,17,18]. Surgical removal with wide margins remains the primary treatment due to the tumor’s aggressive behavior.

## 3. Case Presentation

### 3.1. Case 1

**Figure 1:**
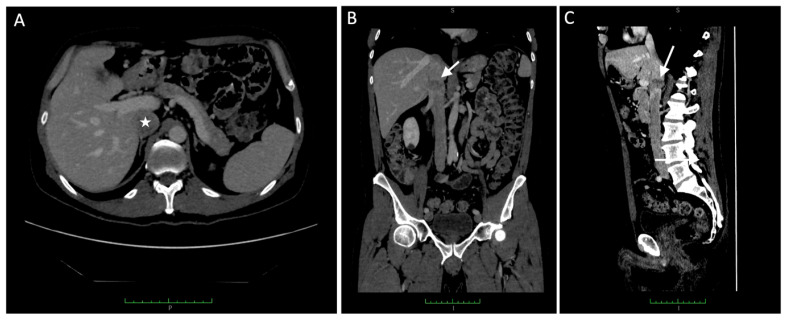
Contrast-enhanced computed tomography of the upper abdomen reveals a heterogeneously enhancing soft tissue mass originating from and expanding the lumen of the inferior vena cava (IVC), as observed on (**A**) axial section (with asterisk), (**B**) coronal section (with white arrow), and (**C**) sagittal section (with white arrow).

A 54-year-old male with a history of ileal loop volvulus caused by an adhesive band underwent surgical treatment one year prior to the current presentation. During that admission, an incidental thrombus-like lesion was identified within the adrenal segment of the IVC, and postoperative anticoagulation therapy was initiated. Follow-up computed tomography angiography (CTA) revealed a heterogeneously enhancing soft-tissue mass arising from and expanding the IVC lumen over an approximate length of 1.5 cm (Figure 1). The lesion contained endoluminal material with abnormal contrast enhancement, raising suspicion of a primary venous sarcoma and prompting further evaluation with F-18 fluorodeoxyglucose positron emission tomography (F-18 FDG PET).

The PET scan demonstrated intense metabolic activity at the lesion site. Histopathological confirmation was not available at this stage. Given the diagnostic uncertainty and the potential oncologic implications, the patient was referred to our tertiary care center for comprehensive diagnostic and multidisciplinary management.

**Figure 2:**
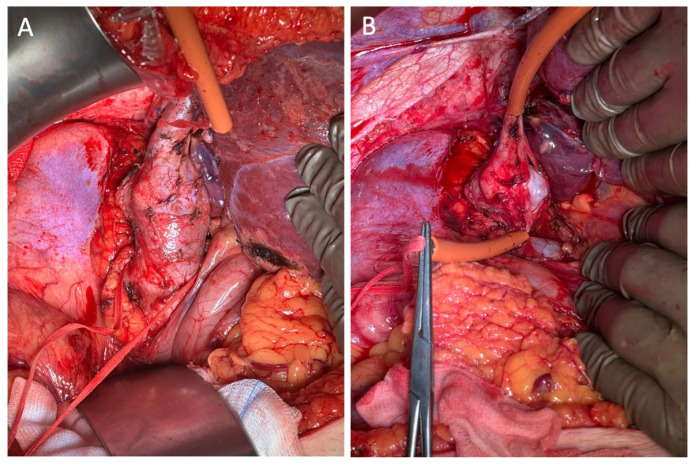

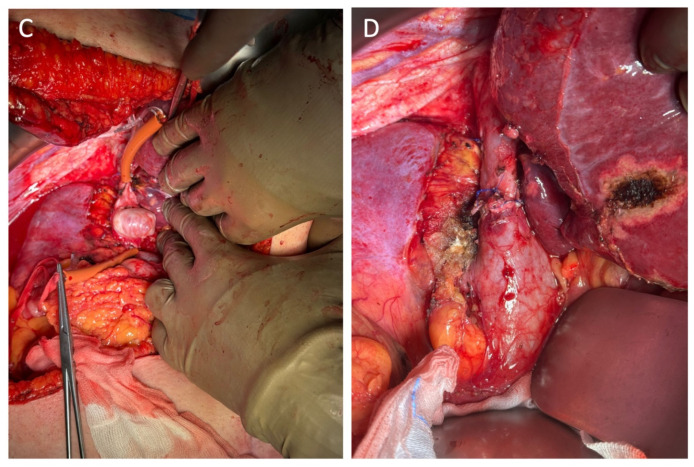
Intraoperative findings demonstrate (**A**) the aspect of the supra-renal IVC segment with proximal and distal vessel loops; (**B**,**C**) endoluminal view of the inferior vena cava exposed via a longitudinal incision, revealing a gray, nodular tumor within the vessel lumen, originating from the vessel wall and consistent with leiomyosarcoma; and (**D**) the direct end-to-end anastomosis of the IVC performed without the utilization of any prosthetic material subsequent to tumor resection.

The chosen treatment was surgical resection, comprising the segmental excision of the affected venous segment followed by a termino-terminal anastomosis (Figure 2). This procedure was executed without the utilization of a vascular prosthesis, thereby establishing vascular continuity via direct end-to-end suturing. The excised specimen was subsequently subjected to histological examination, which revealed findings consistent with a diagnosis of high-grade leiomyosarcoma, a malignancy of smooth muscle origin characterized by spindle cell morphology, significant mitotic activity, necrosis, and infiltrative growth. The proximity of the neoplasm to the surgical margin highlights the necessity for meticulous clinical correlation and the consideration of additional management strategies to ensure local disease control.

### 3.2. Case 2

A 71-year-old female patient presented to our clinic with a painless swelling localized to the left thigh. Her past medical history was notable for mitral and tricuspid valve insufficiency, arterial hypertension, and congestive heart failure. The patient reported that the swelling had gradually developed over the preceding several months. A venous duplex ultrasonography performed eight months prior revealed venous insufficiency with thrombosis of the GSV. On physical examination, a skin-colored, non-tender, and non-pulsatile swelling was observed over the medial aspect of the left thigh. Vital signs were within normal limits. Laboratory investigations, including routine blood tests, were unremarkable. The patient reported no other significant comorbidities or history of surgical interventions.

**Figure 3:**
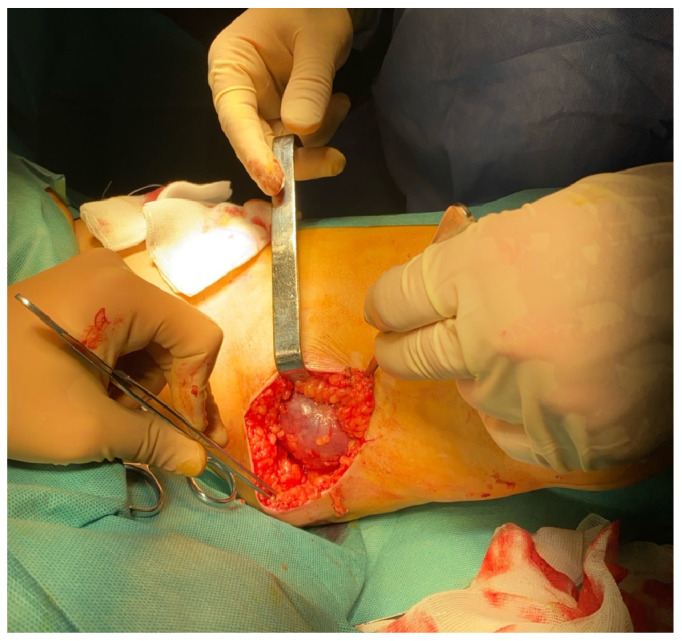
Intraoperative findings revealed a solid dilation of the GSV. The mass is well-demarcated and does not invade surrounding tissues.

Palpation identified a non-tender, mobile mass on the medial surface of the thigh, which appeared superficially mobile despite apparent adherence to deeper anatomical structures. Neurovascular examination of the affected limb demonstrated intact arterial pulses along with preserved motor and sensory function. Considering the progressive enlargement of the lesion and the associated discomfort, surgical excision of the mass, accompanied by ligation of the sapheno-femoral junction and staged stripping of the GSV, was scheduled. Intraoperatively, a firm, partially encapsulated mass measuring 12.2 × 8 × 1.4 cm was identified, originating from the GSV. The mass was entirely excised, followed by ligation at the sapheno-femoral junction and staged stripping of the GSV—performed in an anterograde manner from the premalleolar level to the site of venous tumor formation, and subsequently from that level to the sapheno-femoral junction (Figure 3).

**Figure 4:**
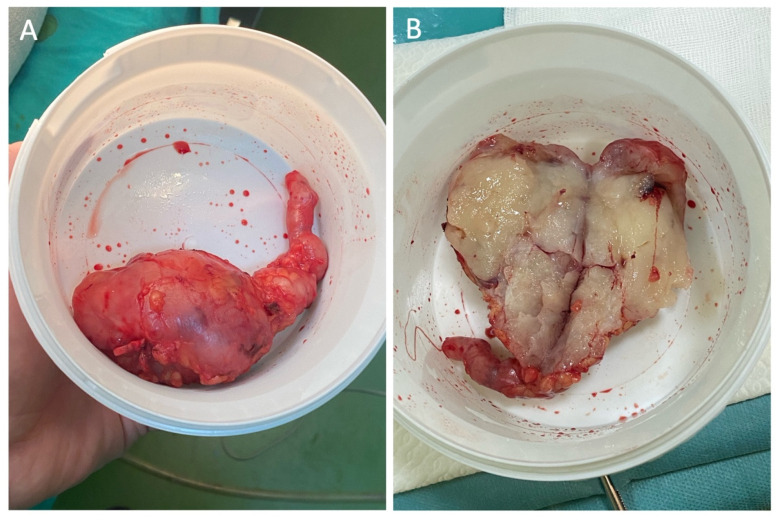
A solid, lobulated mass with an irregular surface and firm consistency, representing the resected tumor: (**A**) shows the external view, and (**B**) shows the cross-sectional surface.

Complete excision with wide margins was achieved, with subsequent GSV stripping (Figure 4). Histopathological evaluation confirmed the diagnosis of LMS arising from the GSV. The tumor exhibits malignant spindle-cell proliferation, arranged in intersecting fascicles that arise from and infiltrate the vascular wall, with elevated mitotic activity (14 mitoses per 10 high-power fields). Immunohistochemical analysis reveals diffuse cytoplasmic positivity for smooth muscle actin, desmin, vimentin, and H-caldesmon in the neoplastic cells, confirming smooth muscle differentiation. Negative staining for CD34 and S100 rules out endothelial and neural differentiation. Ki-67 proliferative index was assessed at 20%.

Both patients reported satisfactory postoperative recovery and expressed understanding of the rarity and severity of their condition. They reported no major functional limitations following surgery and agreed to continue regular oncologic follow-up.

## 4. Discussion

A narrative literature review was performed to identify published reports on leiomyosarcomas involving major venous structures, with particular focus on the inferior vena cava and the great saphenous vein. The search was conducted using the PubMed and Scopus databases. Articles published in English between January 2000 and January 2025 were considered. The following keywords and combinations were used: “leiomyosarcoma”, “inferior vena cava”, “great saphenous vein”, “vascular leiomyosarcoma”, and “venous sarcoma”. Reference lists of relevant articles were also manually screened to identify additional eligible studies. Only clinical reports, case series, and review articles describing primary venous leiomyosarcomas were included (Table 1).

For primary GSV leiomiosarcomas reported after the year 2000, tumor sizes ranged widely from sub-centimetric lesions (1.2 × 0.8 × 0.8 cm) to bulky masses exceeding 10 cm, with most cases clustering between 3–7 cm in diameter. Diagnosis was generally achieved through surgical exploration and histopathological confirmation, with imaging (ultrasound, CT, or MRI) contributing to initial detection in several reports. The therapeutic mainstay across virtually all cases was complete surgical excision, often requiring en bloc resection of the involved venous segment and, in some instances, adjacent soft tissues. Adjuvant measures—particularly radiotherapy and antimitotic chemotherapy—were inconsistently applied, frequently dictated by tumor size, grade, and patient preference.

Complete tumor excision, aiming for R0 resection, is critical to optimizing patient outcomes and reducing the risk of local recurrence. Given the aggressive nature of high-grade tumors and the potential for vascular invasion, adjuvant therapy is often considered [19,20,21,22]. Adjuvant radiation therapy is typically recommended for cases with positive or close surgical margins or when the tumor exhibits high-grade histology to improve local control [23]. Chemotherapy, although its role remains controversial due to limited clinical data, may be employed in advanced, metastatic, or unresectable disease, with regimens commonly including doxorubicin, gemcitabine, and docetaxel. However, adjuvant therapy might yield better results in tumors measuring more than 5 cm [24,25]. A case review published in 2020 reported that leiomyosarcomas originating from the great saphenous vein demonstrate superior five-year survival rates, ranging between 80% and 90%, compared to other large vessel leiomyosarcomas. Tumor recurrence most commonly occurs within 2 to 3 years following surgical intervention [25].

Despite the lack of large-scale clinical trials specific to GSV LMS, evidence extrapolated from soft tissue sarcoma management suggests that combined-modality treatment may enhance disease-free survival, particularly in high-risk patients [1,23]. For primary IVC LMS, surgical options range from segmental resection of the affected IVC segment to more complex procedures, such as ex vivo liver resection and autotransplantation (ELRA), with subsequent IVC reconstruction [10]. Reconstruction of the IVC is commonly performed using prosthetic grafts, such as ringed polytetrafluoroethylene (PTFE), or autologous grafts to restore venous continuity and maintain caval flow. However, ligation may be considered in some cases with adequate collateral circulation [26]. Adjuvant therapies, including chemotherapy and radiotherapy, have unclear efficacy, with doxorubicin-based chemotherapy regimens being the most frequently used but yielding limited response rates. Recently, novel immunotherapeutic approaches combining PD-1 inhibitors, radiotherapy, and granulocyte-macrophage colony-stimulating factor (PRaG therapy) have demonstrated partial disease control in patients with recurrent or metastatic disease [27,28]. Despite aggressive surgical management, high rates of local recurrence and distant metastasis persist, underscoring the need for tailored postoperative surveillance and adjunctive treatments.

**Table 1:** Summary of literature review regarding the GSV LMS cases from the last 25 years.

Year	Source	Tumor Size (cm)	Therapy/Adjuvant	Follow-Up/Outcome
**2004**	Le Minh et al. [19]	1.5 × 2 cm	Wide excision, wide adjuvant therapy, antimitotic agents	Patient is alive, no metastasis present.
		5 × 3 cm low grade sarcoma	No surgical treatment, only adjuvant radiotherapy	6 month postop patient is alive with no metastasis present
**2004**	Marle et al. [20]	3.6 cm diameter	Wide excision; adjuvant radiotherapy refused by patient	2 months postop follow up—patient is alive with no metastatis present
**2006**	Zhang et al. [29]	2 × 4 cm	Wide excision and venous resection	8 months postop follow up—patient is alive and no metastasis present
		3 × 6 cm		
**2006**	El Khoury et al. [21]	6.2 × 9.1 × 10 cm	Wide excision with vein removal, radiation adjuvant therapy and antimitotic agents	No follow up details
**2008**	Mammano et al. [30]	6 cm diameter	En-bloc excision with sartorius muscle and common femoral vein	Liver metastasis 25 months postop with subsequent death 5 months after
**2013**	Werbrouck et al. [31]	6 cm diameter	Wide excision	Bilateral lung metastasis already present at surgery
**2013**	Amato et al. [14]	2 × 1.5 cm	Incomplete excision, follow up surgery with complete excision and vein removal	No metastasis present at follow up
**2013**	Fremed et al. [32]	4 cm diameter	Wide excision and femoral vein removal	No follow up details
**2016**	Lin et al. [22]	20 × 9 mm	Wide excision followed by split skin graft	Unspecified in abstract
**2017**	Cangiano et al. [33]	5 cm diameter	Surgical excision; discussed adjuvant options	Not specified in abstract
**2018**	Macarenco et al. [18]	6.7 cm diameter	Surgical excision	Not specified in abstract
**2019**	Naouli et al. [34]	Not specified in abstract	En-bloc excision with concerned venous segment	Not specified in abstract
**2020**	Fairbairn et al. [25]	6.2 × 9.1 × 10 cm	En-bloc resection with saphenous vein ligation and removal	No follow up details presented
**2020**	Güner et al. [35]	No data available	Surgical resection and adjuvant chemotherapy	No data available
**2021**	Tresgallo-Parés et al. [36]	4 × 2.5 cm	En-bloc excision reported, radiation and antimitotic adjuvant therapy	No follow up details presented
**2022**	Alkhaled et al. [15]	1.2 × 0.8 × 0.8 cm	Surgical excision	No metastasis on staging imaging at presentation; follow-up not extensive
**2022**	Dziekiewicz et al. [37]	Approx. 5 cm diameter	Surgical excision with oncological margins	No metastasis upon follow up examination
		Approx. 10 cm diameter		
**2022**	Fu et al. [38]	10 × 8 cm	Surgical excision and resection of saphenous vein	No follow up information available
**2022**	Irsara et al. [39]	No data available	Surgical excision	No recurrence at 18 month follow up
**2023**	Atieh et al. [40]	6 cm diameter	Surgical resection	Bilateral lung metastasis discovered at follow up
**2025**	Liu et al. [6]	5.9 × 3.6 × 4.5 cm (ultrasound) clinical 6 × 4 cm	Radical surgical resection; later adjuvant chemotherapy for liver metastasis	Liver and lung metastases at 10 months; alive at 13-month follow-up
**2025**	This case	12.2 × 8 × 1.4 cm	Complete excision with wide margins	Patient has been referred to an oncologist at present time.

### Surgical Management Strategies for LMS Involving Major Venous Structures

A comprehensive literature review of IVC LMS cases published up to 2015 was conducted by Watchel et al. [12], encompassing 366 patients. Nearly half underwent prosthetic graft reconstruction after IVC resection, while 21.9% received primary repair and 20.3% received IVC ligation. Reported long-term outcomes showed disease-free survival (DFS) of 57% at 1 year and 57% at 5 years. Overall survival (OS) was 92% at one year, declining to 55% at five years after surgery [12]. Regarding surgical management of IVC LMS, a wide range of techniques has been described, largely determined by tumor location and the extent of venous involvement. In patients with localized disease, primary repair and reconstruction have been reported [2,41]. When the tumor involves only a single caval wall, partial resection followed by reconstruction with an autologous patch [42,43] or with prosthetic materials such as bovine pericardium or a PTFE patch has been successfully performed. In more extensive cases requiring long-segment IVC resection, reconstruction is most often achieved using a PTFE graft [13,44,45,46]. Additionally, Lazat et al. [47] reported a case series using a Dacron graft for IVC reconstruction, with favorable mid- and long-term outcomes. Several authors have also supported IVC ligation without reconstruction after tumor resection as a viable option [2,45,46,48]. Studies by Kieffer et al. [2], Alkhalili et al. [43], Slimane et al. [45], and Zhou et al. [46] suggest that IVC ligation can be safe and does not adversely affect patient outcomes or quality of life, particularly when the LMS has already occluded the IVC and collateral circulation is well established. In contrast, when the tumor is intraluminal, subocclusive, and adherent to a single wall, resection may be limited to the involved wall segment, with subsequent IVC reconstruction using patch techniques [41,42]. In a long-term follow-up study, Teixeira et al. [49] evaluated outcomes in 7 patients who underwent radical IVC resection: 3 underwent IVC ligation without reconstruction, 3 received reconstruction with a Dacron graft, and 1 underwent distal ligation with proximal reimplantation of both renal veins. The authors reported no differences in mortality between the groups. Ultimately, tumor location, the length of IVC resection required, and the presence of adequate collateral venous drainage guide the decision to reconstruct the IVC and determine the most appropriate method. Based on these considerations, we strongly recommend avoiding IVC reconstruction for type I and II lesions when collateral venous return is sufficient. However, when reconstruction is necessary, a PTFE graft prosthesis should be the preferred option.

Primary peripheral LMS are exceedingly rare, and the available evidence is limited to case reports, small case series, and very small patient cohorts [50,51,52,53]. Nevertheless, surgical management generally follows principles similar to those applied in other venous LMS. In a series reported by Sheaffer et al. [50] involving 16 patients, tumor involvement most commonly affected the common femoral vein (CFV) in 10 cases, followed by the iliac vein in 4 patients, and the saphenofemoral junction in 2 cases. Regarding vascular reconstruction, graft interposition was performed in 5 patients, patch angioplasty in 3, and primary venous ligation in half of the cohort [50]. In contrast to traditional open surgical approaches for leiomyosarcoma (LMS), Berelavichus et al. [51] described a two-stage robotic technique for iliac vein LMS. This minimally invasive strategy may facilitate faster postoperative recovery and potentially enhance overall treatment outcomes.

Despite complete resection, leiomyosarcoma carries a clinically meaningful risk of post-treatment local recurrence and distant metastasis [54,55,56,57,58]. Local relapse may necessitate repeat resection and is associated with progressive morbidity, while distant metastasis—most commonly pulmonary—remains the major determinant of disease-specific mortality [54,55,56,57,58]. Therefore, recurrence and metastatic progression significantly reduce overall survival and justify prolonged surveillance following initial treatment.

## 5. Conclusions

In conclusion, vascular leiomyosarcomas affecting the IVC and the GSV are rare cancers that pose unique clinical and surgical challenges. The cases presented highlight the critical importance of early detection and obtaining a definitive histopathologic diagnosis to determine the best treatment approach. The main treatment remains complete surgical removal with negative margins, which greatly impacts prognosis and local disease control. Although adjuvant therapies might offer additional benefits for select high-grade or large tumors, their efficacy remains unclear and warrants further research. This series, combined with an extensive review of the existing literature, underscores the need for a multidisciplinary strategy that considers tumor location and extent, aiming to achieve oncologic clearance while preserving vascular function. Ongoing research into adjuvant and innovative systemic therapies is vital to enhance survival rates and minimize recurrence in this rare subset of vascular sarcomas.

## Data Availability

Any data relevant to this case that are not presented in this manuscript can be obtained from the corresponding author upon reasonable request.

## Abbreviations

The following abbreviations are used in this manuscript:

LMS	Vascular leiomyosarcoma
STS	Soft tissue sarcomas
IVC	Inferior vena cava
GSV-LMS	Great saphenous vein leiomyosarcomas
